# Patient and public involvement in health research from researchers' perspective

**DOI:** 10.1111/hex.13853

**Published:** 2023-08-21

**Authors:** Toril B. Røssvoll, Jan H. Rosenvinge, Kristin Liabo, Tove A. Hanssen, Gunn Pettersen

**Affiliations:** ^1^ Department of Health and Care Sciences Faculty of Health Sciences, UiT The Arctic University of Norway Tromsø Norway; ^2^ College of Medicine and Health, PenCLAHRC Patient and Public Involvement Team University of Exeter Medical School Exeter UK

**Keywords:** occupational therapist, participation, patient and public involvement, reflexive thematic analysis

## Abstract

**Background:**

Patient and public involvement (PPI) is increasingly considered an integral part of health research, and the focus has shifted from *why* we need PPI to *how* users can be involved in a meaningful way. The rationale for investigating experiences with PPI from the perspective of occupational therapy (OT)‐trained researchers' originates in the interrelationship between the inclusive approach to knowledge production, and participation and inclusion as core tenets of OT. The aim of this study was to explore PPI in health research from the perspective of OT‐trained researchers.

**Method:**

Semi‐structured individual interviews were conducted online with nine Norwegian researchers. The interviews were analysed using reflexive thematic analysis.

**Results:**

Professional background and clinical experience from person‐centred OT formed the foundation for how these researchers approached and facilitated PPI in their research. Valuing experiential knowledge and facilitating PPI to be meaningful for public collaborators were highlighted as essential for PPI to have an impact. The need to balance mutual expectations, requirements for research, and what might be possible to achieve within a research study were found to be vital.

**Conclusion:**

Collaborative clinical experience constituted a sound foundation for implementing PPI in research. The occupational perspective underlines the importance of acknowledging experiential knowledge as essential to facilitating meaningful PPI. Challenges related to requirements for research and culture for implementing PPI were addressed by clarifying roles and expectations.

**Patient or Public Contribution:**

Three public collaborators were involved in developing the aims, the interview guide, and the data analysis. They all had previous experience being involved in research.

## 1 INTRODUCTION

Patient and public involvement (PPI) means research being carried out ‘with’ or ‘by’ members of the public, rather than ‘to’, ‘about’ or ‘for’ them.^1^ The term ‘public collaborator’ is used here when referring to patients and members of the public involved in research.^2^

PPI is a requirement for research funding applications and has become an expectation in health research.^3^ Moreover, there has been a growing interest and understanding over the last two decades of the potential for PPI to increase the relevance and quality of health research. One main argument for PPI is people's right to have a say in research where the implications can affect their treatment or lives. A second argument is that PPI provides a useful learning arena with a potential to grasp new ideas with respect to research questions or the interpretation of data, that may enhance research validity and relevance.^4^, ^5^, ^6^, ^7^

There has been a gradual shift of focus from *why* to *how* we can involve patients and the public in health research. Researchers' experiences and attitudes towards PPI, institutional culture and research support, besides an inclusive environment and a safe space for collaboration, are found to be key factors in enabling successful PPI.^8^, ^9^ Barriers have been related to limited funding, time and energy as well as power, ownership and the formation of the research process.^9^, ^10^ To address some of the barriers to PPI, it is important to strengthen the knowledge underpinning its value.^11^

The term ‘participation’, used in this article, has its equivalence in ‘involvement’, notably in life situations,^12^ which converges with core aspects of occupational therapy (OT). Occupational therapists represent a person‐centred health profession concerned with promoting health and well‐being through occupation, where the primary goal is to enable people to participate in the activities of everyday life.^13^ From an occupational perspective, the subjective and social dimensions of participation are central,^14^, ^15^ and require dialogue, collaboration, motivation and the sensitivity of mutual significance.^16^

Person‐centred practice is a central principle in OT, and it has been argued that OT researchers should embed person‐centredness in research to develop knowledge that is relevant to patients' lives, values and priorities.^17^, ^18^ Two OT journals^19^, ^20^ and three recent scoping reviews^21^, ^22^, ^23^ encourage OTs to contribute to the further evolution of PPI by initiating, evaluating and reporting PPI. In Norway, some OT‐trained researchers have had a key role in putting PPI on the agenda within various health research areas, reflecting the congruence between the inclusive approach to knowledge production and the core tenets of OT.^22^

The rationale for investigating experiences with PPI from the perspective of OT‐trained researchers originated in our interest in the interrelationship between the conceptual foundation for OT and PPI described above. The aim of this study was to investigate PPI in health research from the perspective of OT‐trained researchers. The research questions posed were: (1) what constituted the foundation for implementing PPI and (2) how did the OT‐trained researchers approach PPI and address any challenges associated with it?

## 2 METHODS

### 2.1 Design and terminology

We used a qualitative approach with individual interviews as it is suitable to understand the experiences and perceptions of individuals within complex social environments.^24^, ^25^ With the overall aim of enhancing the relevance and quality of the research, three public collaborators were invited to join the research group. In this study, the term ‘PPI group’ refers to academic researchers and public collaborators working together. The PPI group had quarterly meetings, with discussions contributing to learning for all members. All the public collaborators had previous experience of being involved in research, and for this project, two of them got an hourly allowance, while one had the option of using time within her ordinary job.

Research questions and refinements of terminology were agreed upon in the group, and the term ‘public collaborator’ was chosen based on the roles of the public collaborators in this study.

### 2.2 Recruitment

Participants eligible for the study were OT‐trained researchers in Norway with a PhD and experience with PPI in planning and/or performing a health research project. A list of OTs with a PhD was available at the Norwegian Association of Occupational Therapists webpage.^26^ The number of active researchers with PPI experience was not known, although it was lower than 44, which was the number of people on the list at the time of recruitment. Information about the study, along with a request to distribute the invitations to potential participants, was sent to the respective universities and university hospitals where the OT‐trained researchers were employed.

### 2.3 Data collection

The first author conducted the individual interviews online, and the use of M365 Teams were found appropriate for ease of access and cost‐effectiveness. All participants had previous experience of virtual meetings, and the use of video ensured a visual connection between the interviewee and interviewer. We used a semi‐structured interview guide highlighting descriptions of how PPI was implemented, positive experiences, challenges and reflections considering their OT profession in relation to PPI in research. Based on input from public collaborators, questions related to involvement in research dissemination were added to the interview guide.

### 2.4 Demographics of participants

Nine female researchers accepted the invitation to participate and signed an informed consent statement. They held academic positions as professor, post‐doc and associate professor, had various research experience in terms of years since their PhD (1–15 years), working time allocated to research (<30%–100%) and PPI‐experience (from one to several research projects). Some of the participants were researchers before 2015, when PPI became a requirement for health research grant applications in Norway. They, therefore, recalled experiences from that time of change. All participants had clinical OT experience before their research careers, mostly in areas close to their research area, which were within health service and intervention research.

### 2.5 Data analysis

We analysed the data by using a six‐phase reflexive thematic analytic approach,^27^, ^28^ well suited for the identification of patterns across the data set. To familiarise with the data, the first author transcribed the audio recordings and read through the transcripts several times. The initial coding, both semantic (surface meaning) and latent (interpretative) codes, aimed to capture the diversity of meaning within the data set. Through an iterative process, three of the authors (T. B. R., J. H. R. and G. P.), recoded and reread the data, then grouped together the codes (with data extractions) into potential themes and subthemes. The authors and public collaborators discussed preliminary themes in a workshop where themes were revised, defined and named. The group members' varied experience with the topic of the study enriched the analysis and discussions. The process of coding, developing and revising themes was assisted using NVivo 12 (QSR International). Pseudonyms are used for quotations to maintain the participants' anonymity.

### 2.6 Ethical approval

The study adhered to the Helsinki Declaration and was approved by the Norwegian Agency for Shared Services in Education and Research, ref.nr. 292640. The study rested upon the principles of written, informed consent and the option to withdraw unconditionally from the study until all data were deidentified.

### 2.7 Credibility and reflexivity

Several strategies were used to ensure the credibility and quality of the study. The authors have different professional training (nursing, psychology, sociology and OT) and various experiences from PPI in health research. The public collaborators in this study had PPI experience as service users or relatives of service users, two of them had experience of OT service being provided to a family member. The PPI group's various knowledge and experience added to the reflections on the topic for the study, each of the PPI group members' relationship with the topic,^29^ and the specific applicability of PPI to OT. To improve reporting of PPI, the GRIPP2 reporting checklist short version^30^ were applied (Supporting Information 2).

## 3 RESULTS

Following reflexive thematic data analysis, three themes were established.

### 3.1 Initiating PPI originated from the clinical collaboration

Some of the participants related their interest in collaborating with patients in clinical settings to their professional background as occupational therapists, and positive experiences from collaborating with patients in clinical settings created a foundation for involving public collaborators in research. The familiarity of collaboration in different settings and with people with various needs was underlined as particularly valuable when doing PPI. Janne described her transition from person‐centred OT practise to PPI in research this way:

Something I learned from clinical practice was that you will get nowhere without involving the patients. I saw the significance when patients shared experience, took part in evaluations and planning. So, when I started this research project some years later, it was just natural to involve patients in research.

The occupational perspective of participation was explained as essential across their research careers both in terms of their chosen focus of research and the initiating of PPI, before PPI becoming a requirement for health research grant applications by Norwegian research authorities. The occupational perspective meant that these researchers attached value to doing things jointly, finding solutions through collaboration, and viewing PPI as a way of enabling patients' meaningful involvement in society. The participant's professional background as OTs and the occupational perspective of participation served as a driving force in facilitating PPI so that it was meaningful to the public collaborators. PPI was described as an important aid to bridge the gap between knowledge production and knowledge use, which also made it meaningful for the researchers.

### 3.2 Elevating experiential knowledge is valuable

To acknowledge the value and importance of experiential knowledge was one way to safeguard public collaborators during project meetings. It was pointed out that public collaborators can be unfamiliar with the research arena, and a safe space for collaboration and dialogue could be beneficial. A longer time‐horizon was also described to facilitate collaboration, since it takes time for working relationships to evolve. Assuring the public collaborators that their lived experience is a source of unique knowledge was described as fundamental for conducting PPI in a sustainable manner.

I think it is all about being confident in each other. The meetings should constitute a safe environment, and nobody should be afraid of making a fool of themselves, which is universal for all human beings. They {the public collaborators} should be assured, ‘you and your experience are valued here: That's the reason for you being invited’. (Nelly)

The participants talked about how they aimed to make the public collaborators' involvement in research meaningful by facilitating PPI group meetings in line with the collaborators' motivation and expectations. The reward, when public collaborators stated how they found involvement meaningful, was expressed like this:

…it makes me so happy when I get feedback from people when they feel included. For example, when a public collaborator says, ‘I experienced this as a good meeting because I got to say what I wanted, it felt meaningful’. To get this kind of feedback really excites me! (Monica)

Researchers welcomed public collaborators who were unafraid to speak their minds, and although input was not always feasible, the challenge to explain why was appreciated. The participants reported that input from public collaborators elicited in‐depth discussions, which broadened researchers' perspectives and thus could improve both the research process and its outcome. Any uncomfortable aspects of challenging questions were expressed as being necessary since these served as a reminder to the researcher not to assume they knew all the answers.

Group dynamics and handling this were seen as important to ensure that everyone had a say in PPI group meetings. This is how Monica described it:

As a researcher there are many perspectives to safeguard in the meetings, and there will be dynamics in a group. These dynamics must be handled in a proper way, in the sense that all should get the chance to bring in their perspective and get time allotted for speaking. It differs how easily people take the floor, you know. It has been challenging but also exciting because there is so much exciting happening in these meetings.

The researchers described the value of PPI in multiple ways. One participant expressed how she appreciated the opportunity to design a study in collaboration with people ‘who know exactly where the shoe pinches’. Involvement in the early stages was argued to ensure the relevance of the research project and thus be a key motivation for initiating PPI. Regarding recruitment, public collaborators have helped formulate an information letter. This was considered particularly valuable, in addition to their helping to spread information about the study in user forums. Jenny provided the following explanation on how public collaborators substantially contributed to public dissemination:

I think I can communicate fairly well in writing, but I keep getting lost, so it is very good to have them {public collaborators} with me.

Other impacts recounted by these researchers were changes to data collection tools and ensuring the research was acceptable, realistic and useful for both clinicians and patients.

### 3.3 Balancing expectations, requirements and conditions

It was vital for the participants to strive for a balance between expectations from the public collaborators and the opportunities given by the practical research conditions in terms of time and budget. Thus, a good working alliance required talking through what time of day was best for meetings, where meetings should take place, how much time should be spent outside meetings, and the frequency of PPI group meetings. It was essential to clarify the interests, health situation and other commitments of the public collaborators to reach an agreement on which steps of the research process they could be most meaningfully involved in.

I guess it is easier for the public collaborators if they know why they will be involved, and what is expected. The researcher can suggest how the public collaborator can be involved and ask; What do you think about this? The public collaborator can agree, disagree, or bring in their own suggestions. (Stina)

The implementation of PPI was reported as being highly dependent on funding. The budget was described as essential for determining what level of PPI could be implemented, but not detrimental to the decision to carry out PPI or not.

Basically, it all depends on how much money we have. Sometimes there is money for one person with experiential‐based knowledge to take part in the research process. Other times, there is money for an advisory group to meet throughout the project. (Nelly)

The participants shared examples of ‘lessons learned’ related to clarifying roles and expectations for the involvement of public collaborators in the process of writing scientific articles. Because PPI payments typically appear as an allowance for research meetings, the researchers had been concerned about reimbursement for their working hours as coauthors. It was underlined as important to inform the public collaborators, who complied with the requirements of coauthorship, of the budget conditions. By doing this, misunderstandings could be avoided, thereby also probing the public collaborators' motivation to contribute to the writing of manuscripts.

Based on my experiences, co‐authorship might not be the right thing for everyone. For some, yes, but it depends on what they are capable of, what they have knowledge about, and how they understand the research process. (Stina)

Budget matters were also apparent when including public collaborators in developing research designs and grant applications. One way to counter these challenges was to involve staff from patient organisations. The staff could be employed to represent patients or hold relevant lived experience and be in a position of trust. Early involvement of patient organisations was valuable also in terms of recruitment of public collaborators, ensuring inclusion of the kind of experiential knowledge considered most useful.

If a research budget lacked funding for PPI, participants spoke of the option to apply for additional funding and the need to keep public collaborators well informed about what to expect from such applications. Because of their motivation and enthusiasm, many public collaborators were eager to continue their involvement even when these applications had been rejected. However, PPI, without available funding, created a potential ethical dilemma for the researcher regarding how much time and effort the public collaborator could be asked for.

Depending on the capacity the public collaborators had, of course I was aware that they were not paid, so how much can you ask them for, right? (Janne)

How the payment should take place could also imply challenges. Considering PPI in health research is still a relatively new way of doing research, participants voiced a need for a change in administrative routines at research institutions and for PPI to be better incorporated into the overall conditions and frameworks for research.

## 4 DISCUSSION

This study reports on OT‐trained researchers' experiences with PPI in health research. We found that the person‐centred approach stemming from the researchers' professional background and clinical experience formed the interest, motivation and engagement of PPI in health research. Facilitating involvement as a meaningful activity for public collaborators was explained to increase the impact of PPI. The challenges described were related to balancing expectations and personal possibilities with the needs and requirements of the research study.

The participants reflected on their occupational perspective of participation in relation to PPI, and the findings point to a personal as well as social dimension of involvement. The personal dimension concerns an openness to questions and challenges from public collaborators and skills to facilitate collaboration within the PPI group, while the social dimension comprises the requirements for research and culture for implementing PPI. Previous research has also reported on the importance of similar features with respect to interpersonal and communicative competence when establishing and strengthening the PPI group as an inclusive and safe arena for public collaborators to speak up.^8^, ^31^, ^32^, ^33^ As in person‐centred health care, PPI builds on the premise that everyone's contributions have equal value, which comprises mutual respect for the different knowledge, skills and perspectives.^18^

Our findings show the challenge of balancing the expectations of public collaborators with requirements of the research and conditions framing the project, including budget and administrative routines. This finding aligns with previous studies stating that health research funding is not yet designed to fully support PPI.^31^ OT theories recognise that engagement in meaningful activities may require contextual changes,^34^ and there may be practical and logistical barriers to handle.^35^ No matter which descriptions are provided on how to overcome the economic barriers, it is important to be aware of the entailed ethical dilemma considering time use and compensation. Public collaborators and researchers might have various expectations of PPI^36^ and perceive the impact of PPI in different ways.^37^

In line with increasing requirements for PPI, there is a need for researchers to familiarise themselves with ways of involving people with relevant lived experience in their research. To be attentive to the motivation and expectations of public collaborators promotes meaningful PPI, which potentially increases the impact of PPI^38^ and counters a ‘tick‐box’ approach. We hope our findings will encourage researchers to establish a culture of involvement through a deliberate approach to experiential knowledge where its purpose is clearly agreed with public collaborators.

### 4.1 Strengths and limitations

To our knowledge, no other studies have explored PPI from the perspective of researchers with training in the same health profession, for example OT. Although the number of participants could have been larger, the trustworthiness and credibility of our findings rest on the principle of range of perspectives,^39^ which originated from the participants' varying lengths of research experience and different affiliations. Another strength is that the relevance and validity of this research were solicited through the involvement of public collaborators.

## 5 CONCLUSION

Clinical experience in person‐centred health care may create a sound foundation regarding interest in and skills for doing PPI in health research. The occupational perspective in this study underlines the value of facilitating PPI as a meaningful activity for all involved. To improve the possibility for PPI to have an impact, enhancements to the general research context, including funding structures, are suggested.

## AUTHOR CONTRIBUTIONS

Gunn Pettersen and Toril B. Røssvoll led all aspects of the project. Toril B. Røssvoll conducted the data collection and wrote the first draft of the manuscript. Gunn Pettersen led the conceptualisation and design and supported the data collection. Jan H. Rosenvinge, Kristin Liabo and Tove A. Hanssen were peer researchers and contributed to study design and data analysis. All authors contributed to the study's write‐up.

## CONFLICT OF INTEREST STATEMENT

The authors declare no conflict of interest.

## Supporting information

Supporting information.

Supporting information.

## Data Availability

Research data are not shared. The participants have not provided consent for full transcripts to be made available beyond this study.
