# Supplementary material for: Patient and public involvement in health research from researchers' perspective
Source: Health Expect. 2023 Aug 21;26(6):2525–31. doi: 10.1111/hex.13853 (PMC10632614; doi:10.1111/hex.13853)
Supplement: Supplementary file 1 — Supporting information. [file HEX-26--s002.docx]

Interview guide

# Background information

- Experience as a researcher (years since PhD)
- Main employer
- Position / Title
- Working time allocated to research

# Opening question: Can you please tell me about a research project with patient and public involvement that you have been part of?

Follow up questions:

- What was the rationale for initiating PPI in this project?
- How did you go about involving patients or the public?
- Did you have previous experience with PPI, and how?
- What was your role in this project?
- Why did you choose to tell me about project (x)?
- What do you put in the term PPI?

## Involvement in different phases of the research process

Examples of questions:

- In what part of the research process were the public/patients involved? How were they involved?
- Can you please tell how the public/patients were involved in the dissemination process? Both scientific and public dissemination.

## Practical and organisational frames

Examples of questions:

- Can you please explain the practical and organisational frames? (reimbursement, training, etc.)
- What activities were involved in the PPI, and how did you go about them? (Meetings, frequency, length of collaboration, involvement in the research team, dialogue via teams, email, or phone?)

## Positive experiences and challenges along the way

Examples of questions:

- What positive experiences would you highlight?
- What challenges did you meet?
- What kind of improvements would you suggest?
- What thoughts do you have about PPI in the future?

## How do you make use of your professional background as an occupational therapist in PPI health research?

Examples of questions:

- Can you please share your reflections regarding this?
- What reflections do you have about your role as an OT-trained researcher in collaboration with patients or public in research?
- What kind of ‘OT skills’ can be useful in PPI research?
