# Supplementary material for: Patient and public involvement in health research from researchers' perspective
Source: Health Expect. 2023 Aug 21;26(6):2525–31. doi: 10.1111/hex.13853 (PMC10632614; doi:10.1111/hex.13853)
Supplement: Supplementary file 2 — Supporting information. [file HEX-26--s001.docx]

Supplementary Material

GRIPP 2-Short form (Staniszewska et al., 2017).

| Section and topic | Item | Page no |
| --- | --- | --- |
| 1: Aim | Report the aim of PPI in the study. | 3 |
| 2. Methods | Provide a clear description of methods used for PPI in the study. | 4 |
| 3. Study results | Outcomes – Report the results of PPI in the study, including both positive and negative outcomes. | 4, 5, 6 |
| 4. Discussions and conclusions | Outcomes – Comment on the extent to which PPI influenced the study overall. Describe positive and negative effects. | 13 |
| 5. Reflections / critical perspective | Comment critically on the study, reflecting on the things that went well and those that did not, so others can learn from this experience. | N/A |
